# Second-order topological phases in *C*_4*v*_-symmetric photonic crystals beyond the two-dimensional Su-Schrieffer–Heeger model

**DOI:** 10.1515/nanoph-2021-0762

**Published:** 2022-03-01

**Authors:** Yafeng Chen, Zhihao Lan, Jie Zhu

**Affiliations:** State Key Laboratory of Advanced Design and Manufacturing for Vehicle Body, Hunan University, Changsha, Hunan 410082, China; Department of Mechanical Engineering, Hong Kong Polytechnic University, Hong Kong, China; Department of Electronic and Electrical Engineering, University College London, London, WC1E 7JE, UK; School of Physics Science and Engineering, Tongji University, Shanghai, 200092, China

**Keywords:** inverse design, odd-order band gap, photonic crystal, second-order topological insulators

## Abstract

Second-order photonic topological insulators (SPTIs) with topologically protected corner states provide a unique platform for realizing the robust manipulation of light in lower dimensions. Previous SPTIs proposed in *C*_4*v*_-symmetric lattices are mainly based on the two-dimensional (2D) Su-Schrieffer–Heeger (SSH) model consisting of an even number of sites in the unit cell. Moreover, second-order topological phases within high-order band gaps are rarely explored. Here, we propose a new principle of SPTIs beyond the 2D SSH model, which is realized in *C*_4v_-symmetric lattices consisting of an odd number of sites in the unit cell. The midgap-gap-ratios of these odd-order band gaps, from the first-order to the nineteenth-order with step of two-order, are maximized by the method of topology optimization. Second-order topological phases are successfully created within these sizeable band gaps and highly localized corner states are observed. Our work offers a new route for exploring high-order topological states in photonics and other classical systems.

## 1 Introduction

Photonic topological insulators (PTIs), featured with topologically protected edge states that are immune to defects, offer unique ways for realizing robust light transport [1], [2], [3], [4], [5]. Obeying the conventional bulk-boundary correspondence, kaleidoscopic versions of PTIs have been realized based on various physical mechanisms, e.g., quantum Hall effect [6, 7], quantum spin Hall effect [8], [9], [10] and quantum valley Hall effect [11], [12], [13], [14], [15], [16], [17], [18], [19]. Recently, quantized electric multipole insulators [20] and second-order photonic topological insulators (SPTIs) with unconventional bulk-boundary correspondence have been proposed [21, 22]. Different from conventional PTIs with gapless edge states, SPTIs support gapped edge states and topologically protected in-gap corner states, thus providing new ways to realize robust manipulation of light in lower dimensions. By virtue of tightly localized corner states that are immune to defects, SPTIs have found promising applications in topological cavities [23, 24], topological lasers [25] and nonlinear optics [26, 27].

Crystalline symmetry plays a pivotal role in the underlying physics of SPTIs [28]. Hitherto, the main recipe of SPTIs is based on the method of shrunken/expanded unit cell, which has been studied in lattices with different symmetries, such as, kagome lattice with *C*_3*v*_ symmetry [29], hexagonal lattice with *C*_6*v*_ and *C*_3_ symmetries [30], [31], [32], [33], and square lattice with *C*_4*v*_ symmetry [34], [35], [36], [37], [38], [39], among which square lattice has attracted great intentions as it expands the design space beyond traditional graphene-like structures. The study of corner states in PCs up to now has mainly considered the 2D SSH model [40] in square lattice, which has four bands in the tight-binding description, and the band gap covering all the four bands is trivial for both choices of the unit cell, thus cannot be used to support corner states (note one typically uses the band gap between the first and second bands of the 2D square SSH model to engineer corner states, which limits the size of the corresponding band gap). In this work, we propose PCs with odd-order band gaps (i.e., the number of bands below the corresponding band gap is odd, which is also equal to the number of lattice sites within the unit cell in the tight-biding description) and demonstrate the nontrivial topological features of these odd-order band gaps, which can be made very large to support more localized corner states for practical applications. We would like to note that translating the unit cell of a PC with an even-order band gap by (*a*/2, *a*/2) could not change its topological property, thus highlighting the unique features of these PCs with odd-order band gaps. Our work offers a general guidance for exploring second-order topological phases within sizeable odd-order band gaps.

## 2 Results and discussions

**Figure 1:**
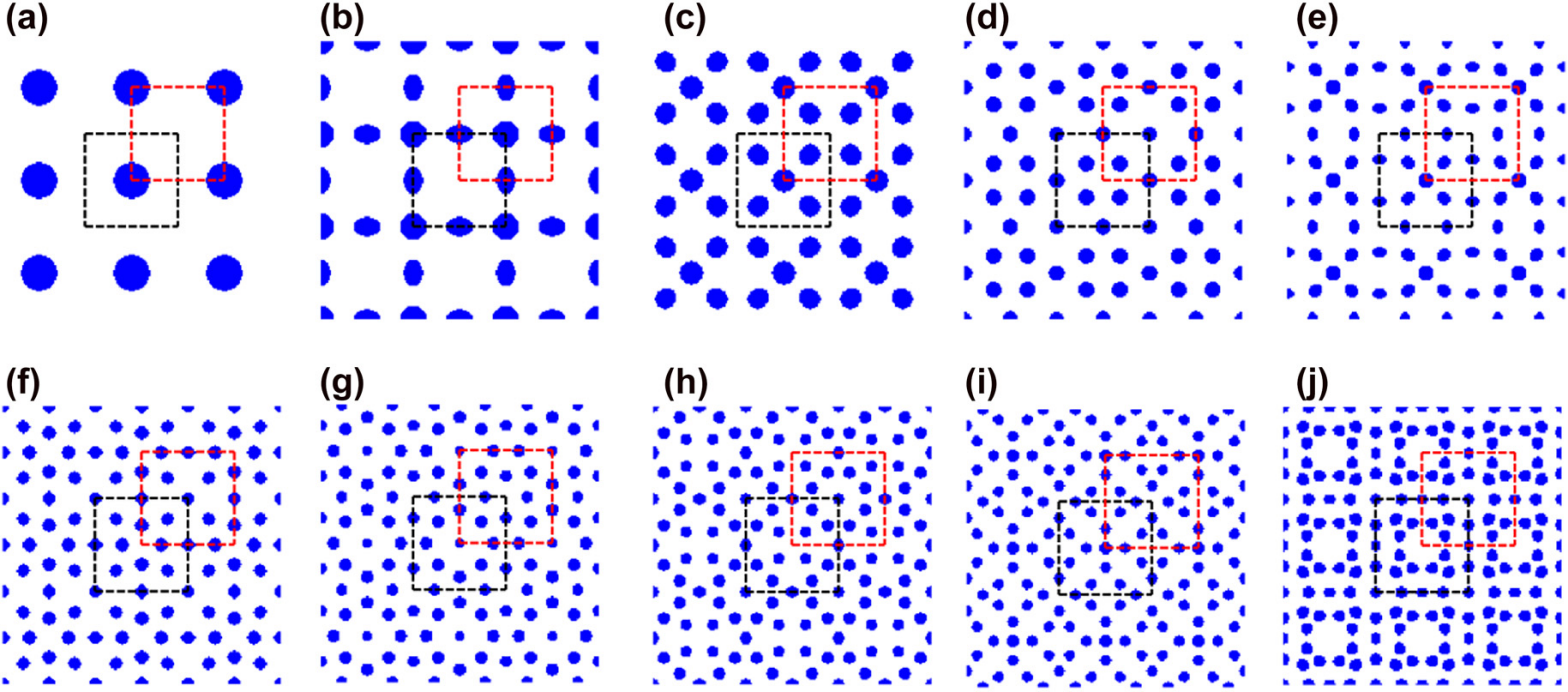
Optimized PCs hosting different odd-order band gaps: (a) the first-order; (b) the third-order; (c) the fifth-order; (d) the seventh-order; (e) the ninth-order; (f) the eleventh-order; (g) the thirteenth-order; (h) the fifteenth-order; (i) the seventeenth-order; (j) the nineteenth-order. The black dashed box denotes the UC*n*A and the red dashed box denotes the UC*n*B.

**Figure 2:**
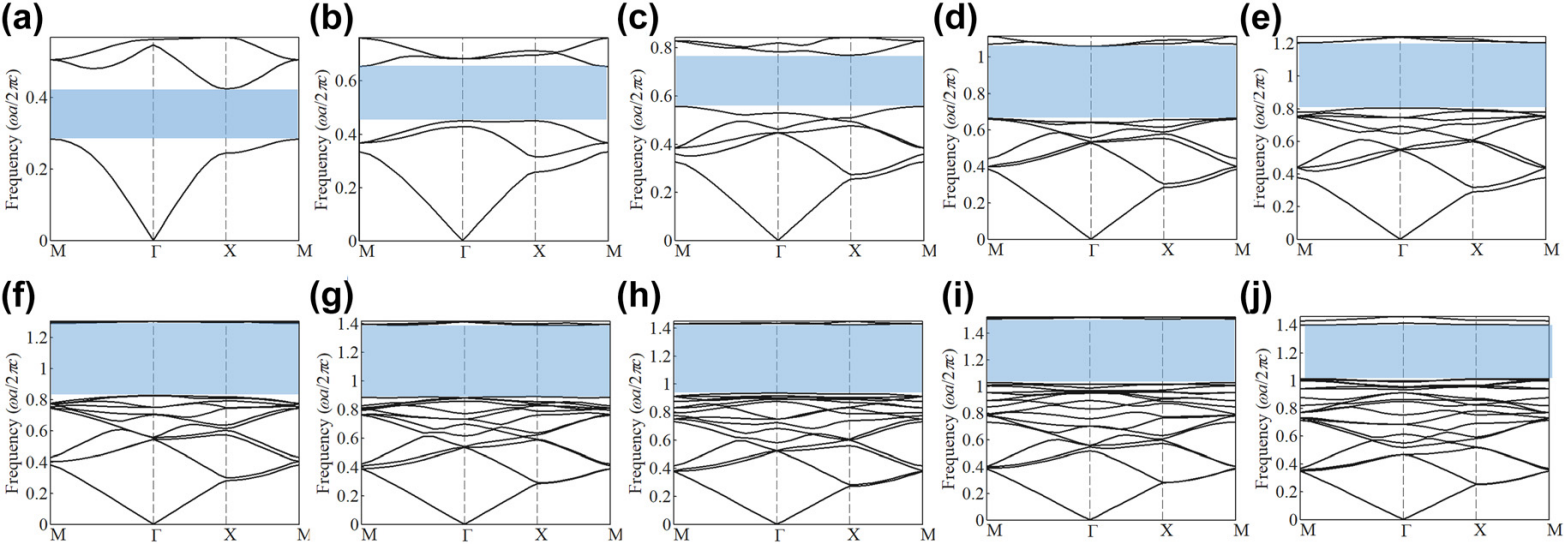
The band diagrams of the optimized PCs hosting different odd-order band gaps (shaded by light-blue regions): (a) the first-order; (b) the third-order; (c) the fifth-order; (d) the seventh-order; (e) the ninth-order; (f) the eleventh-order; (g) the thirteenth-order; (h) the fifteenth-order; (i) the seventeenth-order; (j) the nineteenth-order.

We consider PCs in 2D square lattices that respect the *C*_4*v*_ point group symmetry. The PCs are made of silicon with permittivity of *ε* = 12. Here, we mainly focus on the transverse magnetic (TM) modes while the transverse electric modes can be analyzed similarly. For convenience of discussion, in the following, frequency is normalized with respect to *ωa*/2*πc*, where *ω* is the angular frequency, *a* the lattice constant, and *c* the speed of light. We firstly maximize the odd-order bandgaps (from the first-order to the nineteenth-order) by the method of topology optimization (for details of the optimization method, see Appendix A). Figure 1 shows the optimized PCs (3 × 3 unit cells) with the primitive unit cells denoted by the black dashed boxes, from which one can see that the dielectric materials form isolated dielectric pillars (denoted by blue color) in the optimized PCs. Within the unit cell, the dielectric materials at the internal domain, boundaries and corners of the unit cell form intact pillars, half-pillars, and quarter-pillars, respectively. In practical applications, the pillars, half-pillars, and quarter-pillars can be simplified as cylinders, half-cylinders, and quarter-cylinders, respectively, which can be fabricated by the method of nanoimprint lithography [41]. The number of pillars *n*_p_ within a unit cell can be calculated by *n*_p_ = *n*_i_ + *n*_b_/2 + *n*_c_/4, where *n*_i_, *n*_b_ and *n*_c_ denote the number of pillars at the internal domain, boundaries and corners of the unit cell, respectively. Accordingly, we find that the number of pillars within a unit cell equals to the number of bands below the band gap, which we refer to as the order of the band gap. Note that the number of pillars within a unit cell is odd for all the optimized PCs, which is distinct to the traditional PCs for SPTIs based on the 2D SSH model [34], [35], [36], [37], whose unit cell has an even number of pillars. The band diagrams of the optimized PCs calculated using COMSOL Multiphysics are presented in Figure 2, where the midgap-gap-ratios of the ten optimized PCs are 39.2%, 36.51%, 31.78%, 45.64%, 39.61%, 43.78%, 43.69%, 41.35%, 37.68% and 31.97%, respectively. These extra-wide band gaps significantly exceed those of PCs from the 2D SSH model [34], [35], [36], [37], thus are beneficial to produce more localized edge and corner states. Previous SPTIs mimicking the 2D SSH model indicate that selecting unit cells from the PCs in distinct ways could lead to different topology properties [34], [35], [36], [37]. While our PCs are not based on the 2D SSH model, it is a nontrivial question whether the optimized PCs and these extra-wide band gaps will show any topological properties. To investigate the topological properties of these sizeable odd-order band gaps of the optimized PCs, we choose two different unit cells (UC*n*A and UC*n*B, here *n* is the order of the band gap) from the same optimized PC, where UC*n*A denotes the primitive unit cell of the optimized PC (black dashed box) while UC*n*B (red dashed box) is obtained through translating UC*n*A by (*a*/2, *a*/2). As UC*n*A and UC*n*B encode the same PC, they share the same band diagram. However, the mode shape at high symmetry points (X and Γ) of the first Brillouin zone for each band could be different, which could result in different topology properties between UC*n*A and UC*n*B. The topology properties of UC*n*A and UC*n*B could be determined via the 2D polarization **P** = (*P*_*x*_, *P*_*y*_), defined by [29],

(1) $$P_{i}=\frac{1}{2}\left(\underset{n}{\sum }q_{i}^{n}\text{modulo }2\right),{\left(-1\right)}^{q_{i}^{n}}=\frac{\eta_{n}\left({\text{X}}_{i}\right)}{\eta_{n}\left(\Gamma \right)}$$

in which *i* = *x*, *y* denotes the direction and *P*_*x*_ *=* *P*_*y*_ as the PCs respect the *C*_4*v*_ point group symmetry. *η*_*n*_ denotes the parity of the high symmetry points (X and Γ) for the *n*th band, which could be determined by the eigenmode profile (the *s* and *d* modes have an even parity (+), whereas the *p* mode has an odd parity (−)). The summation over *n* is for all the bands below the band gap. The detailed parity information for all UC*n*As and UC*n*Bs is given in Appendix B. Putting these parities into Eq. (1), we can derive that all UC*n*As are topological trivial, whereas all UC*n*Bs are topological nontrivial. Interestingly, by calculating the number of pillars *n*_*e*_ at one edge of the unit cells ( $n_{e}=\frac{n_{b}^{\prime }}{2}+\frac{n_{c}^{\prime }}{4}$ , where $n_{b}^{\prime }$ and $n_{c}^{\prime }$ denote the number of half-pillars at the boundary and quarter-pillars at the corner, respectively), we find that, if $n_{e}$ is an integer, then the UC is trivial, otherwise the UC is nontrivial. Based on the bulk-boundary correspondence, the distinct topological properties between UC*n*A and UC*n*B ensure the existence of topological edge states at their interface. Meanwhile, the co-existence of nonzero *P*_*x*_ and *P*_*y*_ results in a topological corner charge [42], [43], [44],

(2) $$Q^{c}=4P_{x}P_{y}$$

Accordingly, the corner charges are 0 for UC*n*As and 1 for UC*n*Bs, which predicts the existence of topological corner states at the corner formed between UC*n*As and UC*n*Bs. Note that our numerical experiences indicate that translating the unit cell of PC with even-order band gap by (*a*/2, *a*/2) could not change its topological property. Therefore, we only focus on exploring second-order topological phases with odd-order band gaps in this paper. To capture the key topological features of the optimized PCs in a transparent way, we construct simple tight-binding lattice models based on the configurations of UC*n*As and UC*n*Bs, as given in Appendix C.

**Figure 3:**
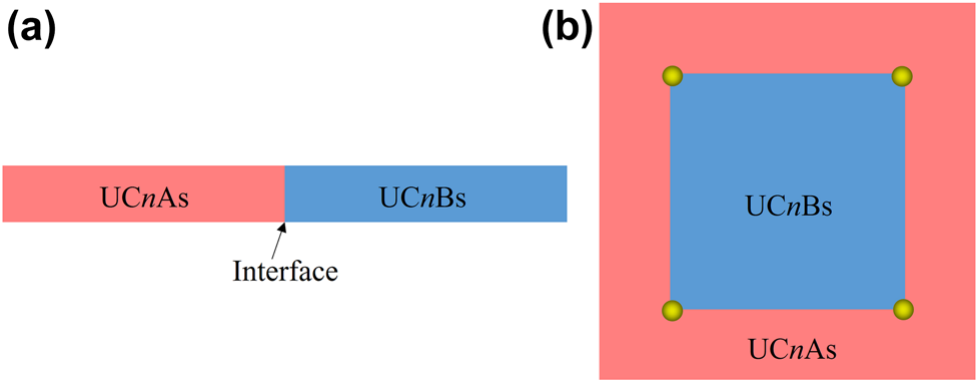
Sketchs of the supercell and the metastructure for the calculation of  (a) Topological edge states and (b) Topological corner states.

**Figure 4:**
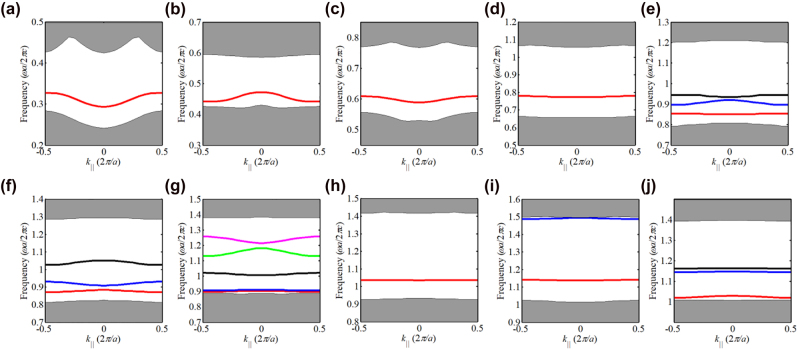
The projected band diagrams of the supercells consisting of (a) UC1A and UC1B, (b) UC3A and UC3B, (c) UC5A and UC5B, (d) UC7A and UC7B, (e) UC9A and UC9B, (f) UC11A and UC11B, (g) UC13A and UC13B, (h) UC15A and UC15B, (i) UC17A and UC17B, and (j) UC19A and UC19B.

To verify the topological edge states, the ribbon structure consisting of 6 UC*n*As and 6 UC*n*Bs with an interface between them is built, as sketched in Figure 3(a). Figure 4 shows the calculated projected band diagrams of the ribbon structures made of PCs with different orders of band gaps, and we can see that edge states appear within the bulk band gaps for all the ribbon structures. Importantly, these edge states are gapped, satisfying the prerequisites to produce corner states. The eigenfield distributions of these edge states are given in Appendix D.

**Figure 5:**
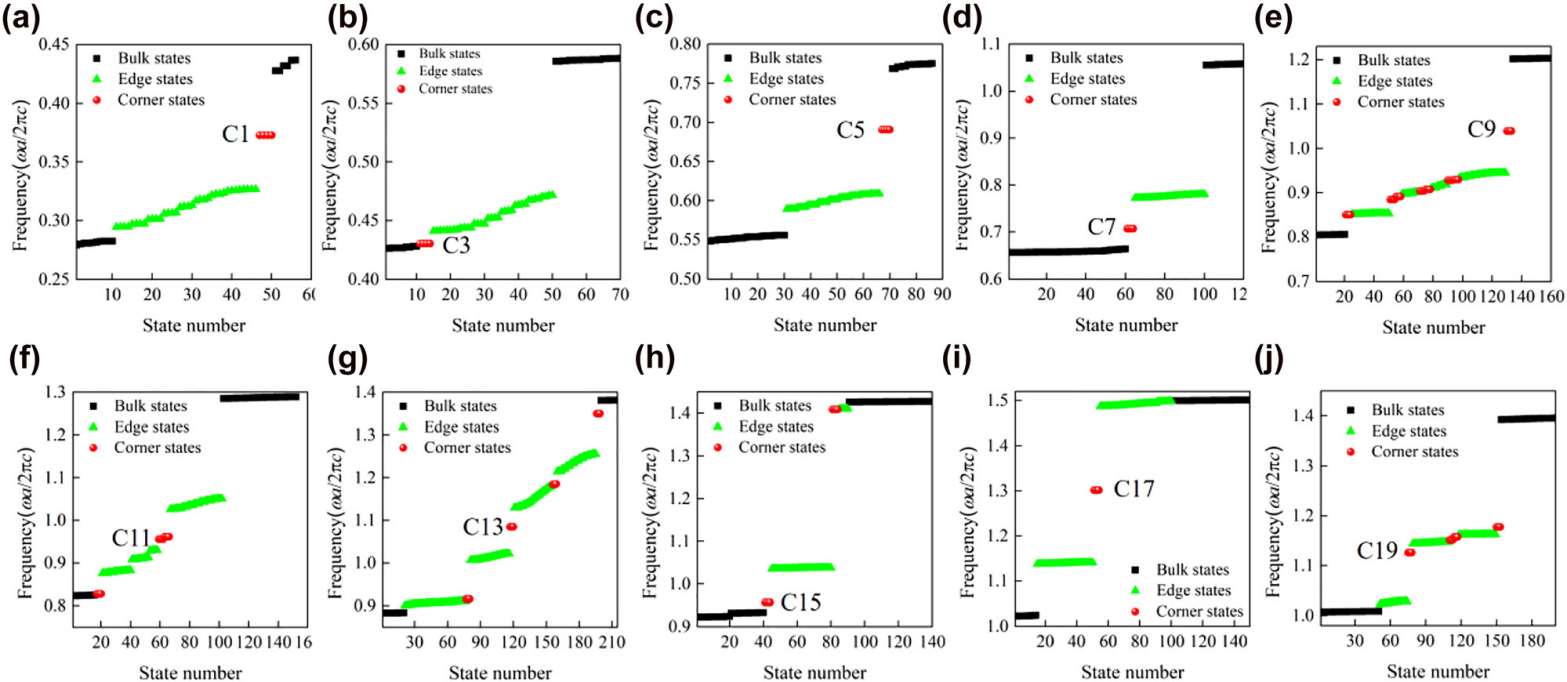
Calculated eigenfrequencies of (a) MS1, (b) MS3, (c) MS5, (d) MS7, (e) MS9, (f) MS11, (g) MS13, (h) MS15, (i) MS17, and (j) MS19. MS*n* represents that the metastructure is made of UC*n*A and UC*n*B.

**Figure 6:**
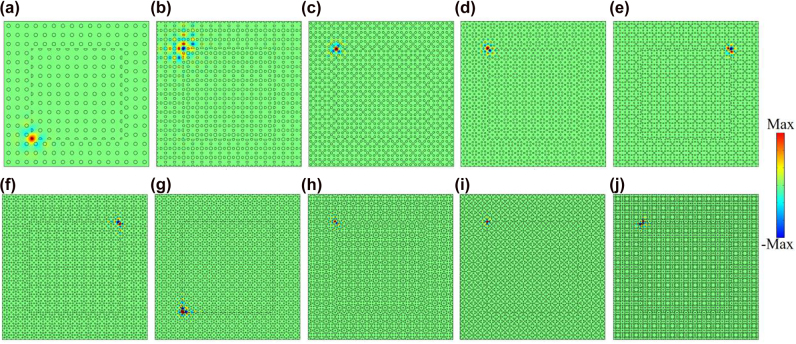
Eigenfield distributions of one representative corner state labelled by C*n* in Figure 5. (a) C1, (b) C3, (c) C5, (d) C7, (e) C9, (f) C11, (g) C13, (h) C15, (i) C17, and (j) C19.

To confirm topological corner states, we construct the metastructure consisting of 10 × 10UC*n*Bs surrounded by 3 layers of UC*n*As, as sketched in Figure 3(b). As a result, the metastructure comprises four corners that can host corner states, as denoted by the yellow balls. We label the metastructure consisting of the optimized PCs with the *n*th-order band gap as MS*n*. Figure 5 shows the calculated eigenfrequencies of MS1-MS19 by using COMSOL Multiphysics. It reveals that MS1, MS3, MS5, MS7, and MS17 host one batch of corner states consisting of four degenerated corner states. However, distinct from current SPTIs mimicking the 2D SSH model, the remaining MSs support multiple batches of corner states, thus providing possibilities for designing novel multiband photonic devices based on corner states. We would like to note that the topological invariant (i.e., Eq. (2)) of the current systems is a binary number (0 or 1) and as such it can only indicate the topologically trivial or nontrivial nature of the band gap, but cannot be used to determine the number of corresponding edge or corner states, which in general depends on the specific details (e.g., shape of the corner [45, 46] or parameters [47]) of the underlying PCs. Figure 6 presents the eigenmodes of corner states labeled by C*n* in Figure 5. We can see that all corner states are indeed localized at the corners of the MSs. Moreover, the wide band gaps resulted from topology optimization enable more localized corner states. To quantitatively compare the localization degree of corner states in Figure 5 with that of corner states based on the 2D SSH model in Reference [35] (labeled by *C*_SSH_), we further compute their mode volumes [48], defined by $V_{m}=\frac{\int \epsilon \left(r\right){\left|E\left(r\right)\right|}^{2}dV}{\max\left(\epsilon \left(r\right){\left|E\left(r\right)\right|}^{2}\right)}$ with *V* denoting the area of MS*i*, and the *Q* factors. The number of trivial and nontrivial UCs for constructing the SPTIs is the same. Table 1 below summarizes the results and it can be seen that, compared to *C*_SSH_, the mode volumes of corner states obtained herein are lower and the *Q* factors are larger, indicating that these corner states are more localized. In particular, the *Q* factors of *C*_5_–*C*_19_ are several orders of magnitude higher than *C*_SSH._

**Table 1:** Mode volumes and *Q* factors of *C*_1_–*C*_19_ and *C*_SSH_.

	*C*_SSH_	*C*_1_	*C*_3_	*C*_5_	*C*_7_	*C*_9_	*C*_11_	*C*_13_	*C*_15_	*C*_17_	*C*_19_
*V*_*m*_	0.482	0.061	0.223	0.021	0.029	0.012	0.020	0.025	0.019	0.006	0.023
*Q* factor	2.6 × 10^2^	3.9 × 10^3^	3.8 × 10^2^	2.3 × 10^6^	2.3 × 10^6^	9.4 × 10^10^	1.6 × 10^11^	1.8 × 10^12^	2.2 × 10^7^	2.5 × 10^13^	8.8 × 10^10^

## 3 Conclusions

In summary, we propose a series of SPTIs hosting sizeable odd-order band gaps in *C*_4*v*_-symmetric PCs beyond the widely used 2D SSH model. The relative sizes of these odd-order band gaps, from the first-order to the nineteenth-order, of the PCs are maximized by the method of topology optimization. The optimized PCs consist of an odd number of pillars in the unit cell and their band gaps significantly exceed those of PCs based on the 2D SSH model. We demonstrated that second-order topological corner states exist within these band gaps, which are more localized than those based on the 2D SSH model. The finding of topological corner states within high- and odd-order band gaps fills the uncharted region of topological photonics. Our work brings new perspectives for engineering high-order photonic topological phases and the principle could be applied to other classic systems as well.

## Figures and Tables

**Figure A1:**
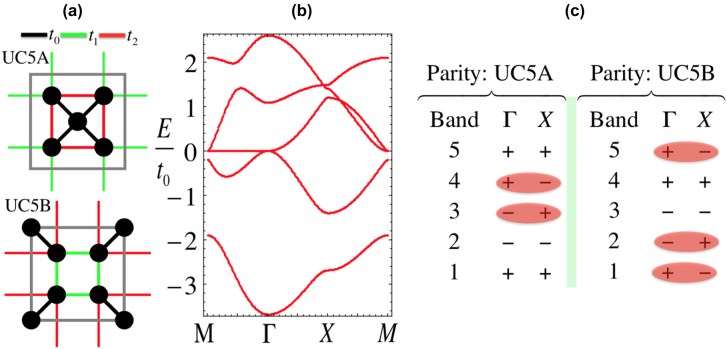
Tight-binding model and results of UC5. (a) The two unit cells UC5A and UC5B and corresponding hopping patterns described by $t_{0,}t_{1},t_{2}.$ (b) The energy band diagram at $t_{0}=1.0,t_{1}=0.7,t_{2}=0.6$ . (c) The parity distributions of the five energy bands at (Γ,X) for UC5A and UC5B.

**Figure A2:**
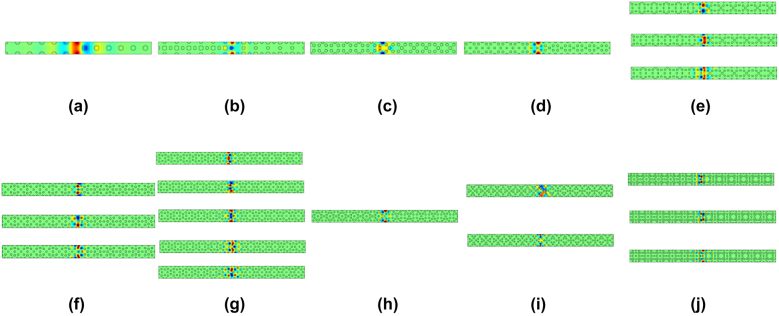
Eigenfield distributions of edge states (*k*_||_ = 0) for the supercells consisting of (a) UC1A and UC1B, (b) UC3A and UC3B, (c) UC5A and UC5B, (d) UC7A and UC7B, (e) UC9A and UC9B, (f) UC11A and UC11B, (g) UC13A and UC13B, (h) UC15A and UC15B, (i) UC17A and UC17B, and (j) UC19A and UC19B.

## Appendix A: The topology optimization approach to maximize the odd-order band gap

For TM modes, the governing equation of electromagnetic waves propagating in photonic crystals (PCs) is [49]

(A1)
$$-\left(\nabla +ik\right)\cdot \left(\left(\nabla +ik\right)E\left(r\right)\right)=\epsilon \left(r\right){\left(\frac{\omega }{c}\right)}^{2}E\left(r\right)$$

in which **r** is the position vector, *E*(**r**) the electric field perpendicular to the propagation plane, *ε*(**r**) the dielectric function, *ω* the eigenfrequency, *c* the speed of light, and **k** = (*k*_*x*_, *k*_*y*_) the wave vector.

The finite element method is utilized to solve Eq. (A1). The domain is discretized as finite elements with each element assigned with design variable *x*_*e*_, and its permittivity is linearly interpolated as

(A2)
$$\epsilon \left(x_{e}\right)=\epsilon_{1}\left(1-x_{e}\right)+\epsilon_{2}x_{e}$$

in which *ε*_1_ and *ε*_2_ are permittivities of air and dielectric material, respectively. Then, Eq. (A1) can be converted to a typical eigenvalue equation as

(A3)
$$\left(K-{\left(\frac{\omega }{c}\right)}^{2}M\right)u=0$$

in which **K** and **M** are global matrixes and **u** is the eigenvector corresponding to the electric field.

The optimization objective is to maximize the odd-order band gap, which can be expressed as

(A4)
$$\text{max}:f=2\frac{\text{min}\omega_{n+1}\left(k\right)-\text{max}\omega_{n}\left(k\right)}{\text{min}\omega_{n+1}\left(k\right)+\text{max}\omega_{n}\left(k\right)}$$

in which *n* denotes the order of the band gap.

As we use the gradient-based optimization algorithm herein, the derivative of *f* with respect to *x*_*e*_, namely, sensitivity, should be derived, which can be obtained upon the calculation of $\frac{\partial \omega_{n}\left(k\right)}{\partial x_{e}}$ and $\frac{\partial \omega_{n+1}\left(k\right)}{\partial x_{e}}$ . Differentiating both sides of Eq. (A3), $\frac{\partial \omega_{n}\left(k\right)}{\partial x_{e}}$ can be derived as

(A5)
$$\frac{\partial \omega_{n}\left(k\right)}{\partial x_{e}}=\frac{1}{2\omega }u^{T}\left(c^{2}\frac{\partial K}{\partial x_{e}}-\omega^{2}\left(k\right)\frac{\partial M}{\partial x_{e}}\right)u$$

After obtaining the sensitivities, we use the method of moving asymptotes (MMA) [50, 51] to iteratively update the design variable to maximize the objective function *f*.

## Appendix B: Parities at Γ and X of the band structures for UC*n*A and UC*n*B

Table A1 presents parities at Γ and X of the band structures for UC*n*A and UC*n*B, where parities with opposite signs at are shaded with blue colors.

**Table A1:** Parities at high symmetry points of the band structures for UC*n*A and UC*n*B.

		The order of band																		
		1	2	3	4	5	6	7	8	9	10	11	12	13	14	15	16	17	18	19
UC1A	Γ	+																		
	X	+																		
UC1B	Γ	+																		
	X	−																		
UC3A	Γ	+	+	+																
	X	−	+	−																
UC3B	Γ	+	+	+																
	X	+	−	+																
UC5A	Γ	+	−	−	+	+														
	X	+	−	+	−	+														
UC5B	Γ	+	−	−	+	+														
	X	−	+	−	+	−														
UC7A	Γ	+	+	−	−	+	+	+												
	X	+	−	−	−	+	+	−												
UC7B	Γ	+	+	−	−	+	+	+												
	X	−	+	+	+	−	−	+												
UC9A	Γ	+	+	−	−	+	+	−	−	+										
	X	+	−	+	+	−	−	−	+	+										
UC9B	Γ	+	+	−	−	+	+	−	−	+										
	X	−	+	−	−	+	+	+	−	−										
UC11A	Γ	+	−	−	+	+	+	+	−	−	+	+								
	X	+	−	−	−	+	+	+	−	−	+	−								
UC11B	Γ	+	−	−	+	+	+	+	−	−	+	+								
	X	−	+	+	+	−	−	−	+	+	−	+								
UC13A	Γ	+	−	−	+	+	+	−	−	+	+	−	−	+						
	X	−	+	+	−	−	+	+	−	+	+	−	−	+						
UC13B	Γ	+	−	−	+	+	+	−	−	+	+	−	−	+						
	X	+	−	−	+	+	−	−	+	−	−	+	+	−						
UC15A	Γ	+	−	−	+	+	+	+	−	−	+	−	−	+	+	+				
	X	−	+	+	−	−	+	−	+	−	+	−	−	+	−	+				
UC15B	Γ	+	−	−	+	+	+	+	−	−	+	−	−	+	+	+				
	X	+	−	−	+	+	−	+	−	+	−	+	+	−	+	−				
UC17A	Γ	+	+	+	−	−	−	−	+	+	+	−	−	+	−	−	+	+		
	X	+	−	+	+	−	−	−	+	+	−	−	+	+	−	+	−	+		
UC17B	Γ	+	+	+	−	−	−	−	+	+	+	−	−	+	−	−	+	+		
	X	−	+	−	−	+	+	+	−	−	+	+	−	−	+	−	+	−		
UC19A	Γ	+	−	−	+	+	+	−	−	+	+	+	−	−	+	+	−	−	+	+
	X	−	+	−	+	−	+	−	+	+	−	−	+	−	+	+	−	−	−	+
UC19B	Γ	+	−	−	+	+	+	−	−	+	+	+	−	−	+	+	−	−	+	+
	X	+	−	+	−	+	−	+	−	−	+	+	−	+	−	−	+	+	+	−

## Appendix C: Tight-binding models of the proposed PCs

The topological properties of the band gaps in Figure 2 and the band structures below the band gaps could be captured by a tight-binding description, in which one can take the dielectric pillars as lattice sites and consider a particle hops in the corresponding lattice according to the following Hamiltonain,

(C1)
$$H=-\sum_{〈ij〉}t_{ij}c_{i}^{†}c_{j}$$

where $t_{ij}$ is the hopping amplitude between lattice sites and $c_{i}^{†}\left(c_{i}\right)$ is the creation (annihilation) operator of the particle at site i . As we consider a particle without any internal degree of freedom, the number of bands in this tight-binding description (i.e., the ones below the band gaps shown in Figure 2) is equal to the number of lattice sites in the unit cell (see Figure 1).

In the following, we illustrate through some examples how the tight-binding description will work. First, we consider the simplest case of Figure 1(a), where there is only one lattice site in the unit cell. In this case, there is only one energy band in the tight-binding model whose expression can be simply worked out,

(C2)
$$E=-t_{0}\left(e^{ik_{x}}+e^{-ik_{x}}+e^{ik_{y}}+e^{-ik_{y}}\right)=-2t_{0}\left(cos\;k_{x}+cos\;k_{y}\right)$$

For UC1A, where the lattice site is at the center of the unit cell, choosing the center of the unit cell as the inversion center, the inversion operator is just I=1 . So the parities at (Γ,X) are simply (+,+) (see also Table A1). For UC1B, where the lattice sites are at the corners of the unit cell, the inversion operator $I=e^{\pm i\left(k_{x}+k_{y}\right)}$ depending on which lattice site is used in the tight-binding description as the four lattice sites at the corners of the square unit cell are equivalent and under inversion operation, the two pairs of lattice sites under the diagonals transform to each other along the diagonals. So at Γ=(0,0) the parity is +1 whereas at X=(π,0) , the parity is $I=e^{\pm i\pi }=-1$ , which agrees with the results in Table A1.

Next, we study a more complicated case of Figure 1(c), where each unit cell has five lattice sites, i.e., there are five energy bands below the corresponding band gap. The results are presented in Figure A1. To match more closely with the energy band diagram in Figure 2(c), three hopping strengths of $t_{0}=1.0,t_{1}=0.7,t_{2}=0.6$ are introduced (the larger the distance between the lattice sites the smaller the hopping strength between the corresponding lattice sites). Comparing the band diagrams in Figure 2 in the main text and Figure A1 below, we can see they show a reasonable agreement considering the fact that much longer hoppings exsit in the full-wave simulations of the real PC. Note that as the two unit cells UC5A and UC5B describe the same lattice, their band diagrams are the same, i.e., the one given in Figure A1. The parity distributions from the tight-binding calculations given in Figure A1 for UC5A and UC5B show perfect agreement with the results from full-wave simulations of the real PC presented in Table A1.

Similar analysis could be performed for the other PCs shown in Figure 1. However, as the number of dielectric pillars within the unit cells increases, the tight-binding formualtions would become increasingly complex, where the full-wave simualtions would be more realistic and straightforward. We would like to note that as the tight-binding lattice models are able to capture the key topological features of the optimized PCs in a general way, the topological physics of the PCs with odd-order band gaps discussed in this paper could carry over to diverse system platforms beyond photonics, such as, plasmonics, acoustics, as long as these systems can execute a faithful implementation of the tight-binding model.

## Appendix D: Eigenfield distributions of edge states

Figure A2 presents eigenfield distributions of edge states (*k*_||_ = 0) in Figure 4. The eigenfield distributions from bottom to top for the multiple edge modes correspond to multiple edge states from bottom to top in Figure 4, respectively.
